# Impact of ketosis on neuronal activity: a characterization of the functional role of ketone bodies using non‐invasive encephalography in humans

**DOI:** 10.1002/alz70856_103513

**Published:** 2025-12-26

**Authors:** Felipe Ignacio González Henríquez, Vicente Medel, Daniel Franco O'Byrne

**Affiliations:** ^1^ Pontificia Universidad Católica de Chile, Santiago, Santiago, Chile; ^2^ Latin American Brain Health Institute (BrainLat), Universidad Adolfo Ibáñez, Santiago, Región Metropolitana de Santiago, Chile; ^3^ Latin American Brain Health Institute (BrainLat), Universidad Adolfo Ibáñez, Santiago, Región Metropolitana, Chile

## Abstract

**Background:**

Neurometabolism plays a critical role in modulating dynamic brain activity through its impact on energy supply. While glucose is the primary energy source for neurons, ketone bodies are emerging as a key alternative, using an astrocytic pathway specially active under under high energy demand states. The ketogenic diet, known for its ability to enhance ketone production, has been suggested to influence cognitive performance and protect the aging brain. However, the neural mechanisms are still unknown.

**Method:**

This study uses an open dataset (van Nieuwenhuizen et al., 2024) to examine the impact of the ketogenic diet on modulating neuronal activity through the modulation of neuronal energy supply, using non‐invasive electroencephalography (EEG). A sample of 36 healthy adults was assessed under two conditions: (1) a ketogenic condition, where participants consumed a ketone ester supplement (395 mg/kg) and (2) a glucogenic condition, where they consumed an equivalent caloric bolus of glucose. Resting‐state EEG data were recorded using a 65‐channel system under both conditions, and preprocessing techniques, including artifact removal and frequency band separation, were applied. Neuronal dynamics were assessed via the spectral analysis of the 1/f aperiodic activity.

**Results:**

Findings indicate significant modulation of EEG aperiodic 1/f activity under the ketogenic condition and not glucogenic condition. Specifically, a flatter 1/f aperiodic slope suggest an enhanced information processing state with increased neuronal neuronal dynamic brain state, similar to a healthy attentive state.

**Conclusion:**

The ketogenic diet enhances neuronal aperiodic 1/f activity by providing a fast and alternative energy source in the form of ketone bodies. These findings suggest potential therapeutic applications for aging and neurodegenerative disorders for ketogenic interventions and suggest a key modulation of the central nervous system, likely involving astrocyte‐neuron pathway.

**Authors**:

Felipe González*, Daniel Franco‐O'Byrne*, Ana María Castro‐Laguardia, Tomás Bosch, Tomás Ossandón, Vicente Medel

*=equally contributing authors °=corresponding author

Funding: FONDECYT Exploración 13240170 and FONDECYT de Iniciación 11251578.

